# The effect of nurse-initiated diary intervention on posttraumatic stress disorder and recall of memories in ICU survivors: a randomized controlled trial

**DOI:** 10.1186/s12888-024-05581-x

**Published:** 2024-02-22

**Authors:** Elham Rashidi, Farideh Razban, Neda Asadi

**Affiliations:** Nursing Research Center, Kerman University of Medical Sciences, Kerman, Iran

**Keywords:** Diary, Post-traumatic, Stress disorder, Memory, Intensive care units

## Abstract

**Background:**

Patients’ recall of memories from the ICU plays an important role in the occurrence of post-traumatic stress disorder. This study aimed to determine the effect of nurse-initiated diary intervention on post-traumatic stress disorder and recall of memories in ICU survivors.

**Methods:**

This RCT study included all patients admitted to two trauma ICUs in Southeast of Iran. Thirty patients considered in control and intervention groups. Data collection tools included the Impact of Events Scale-Revised (IES-R), and the ICU Memory Tool (ICU-MT). The researcher wrote daily diaries of the intervention and control groups during the first 72 h of their admissions. SPSS25 was used to analyze the data.

**Results:**

The total mean PTSD score in the intervention group was significantly lower than that in the control group (*p* > 0.0001, z = -3.75). The number of those in the intervention group who clearly recalled their admission to hospital, their hospital stay before being admitted to the ICU and all memories from the ICU stay, was more than those in the control group; this difference was statistically significant (*p* > 0.0001).

**Conclusion:**

The results showed that the nurse-initiated diary was effective on the PTSD and recall clear memories of patients admitted to the ICU. We suggest medical and educational centers to use this intervention in order to reduce the posttraumatic stress disorder in these patients. As nurse-initiated diary intervention had no significant difference in the recall of different types of memories from the ICU, we require further studies in this field.

## Introduction

As the number of people suffering from serious and life-threatening diseases is increasing in the world, they require special care [1, 2]. Admission to ICU improves the quality of care and minimizes the mortality rate and the consequences related to the disease [3]. Post-intensive care complications include cognitive disorders, acquired weakness, and intrusive memories similar to post-traumatic stress disorder [4]. According to the diagnostic and statistical manual of mental disorders, post-traumatic stress disorder is prolonged complications after experience of severe stressors [5]. Invasive measures, continuous monitoring and uncertainty of full recovery increase PTSD in ICU patients three times more than patients in other departments [6].

ICU survivors may recall stressful memories from the ICU, which may appear in three forms: factual, emotional, and delusional memories. Factual memories are real ICU knowledge acquired in the past rather than through experience or observation in the present. Staff and visitors, ambient noise and lighting, extubation, and tracheal suction are factual memories mentioned by patients in the ICU [7]. Emotional memories refer to emotional stimuli or events that happened in the ICU, including memories of pain, discomfort, confusion, anxiety or fear [8]. A significant number of ICU survivors report delusional memories related to sleep, nightmares, paranoid delusions, and delirium experienced in the ICU [9]. Several stressful memories may lead to post-traumatic stress disorder after discharge [8]. Askari Hosseini et al. showed that 34% of the ICU survivors had delusional memories, 66% had emotional memories, and 89% had factual memories [10].

Nurses play an important role in providing care and preventing ICU complications in patients [8]. Nurse-initiated diary intervention has increased patients’ trust and acceptance in what they read [11]. Nurse-initiated diary intervention provides objective and real information to patients, discard unreal experiences, reconstruct one’s own experiences in the ICU, and improve perception of confusing memories. Using diaries as a way to summarize what happened to ICU, help patients understand the sequence of events that happened in the ICU. The use of diaries in the ICU is a tool to fill the patient’s memory gaps and reduce the occurrence of mental disorders in patients after discharge from the ICU [12]. Torres et al. indicated that the nurse-initiated diary intervention reduced posttraumatic stress in ICU survivors [13], but another study did not support this method to prevent this disorder [12].

Considering the high prevalence of post-traumatic stress and its unpleasant consequences in ICU survivors, we decided to study the effect of nurse-initiated diary intervention on post-traumatic stress disorder and recall of memories in ICU survivors.

## Methods

### Design

This randomized clinical trial study investigated the effect of nurse-initiated diary intervention on post-traumatic stress disorder and recall of memories in ICU survivors. This research was approved by the Ethics Committee of Kerman University of Medical Sciences (1400553IR.KMU.REC.) and was registered in the Iranian Registry of Clinical Trials IRCT20220105053633N1. https://en.irct.ir/trial/6113826/01/2022.

### Sample and setting

We studied patients admitted to two trauma ICUs of Bahoner hospital affiliated to Kerman University of Medical Sciences. Inclusion criteria were patients aged 19-80 years, who stayed in ICU more than 72 h, were able to read and write in Persian, had no severe brain trauma (Glasgow Coma Scale ≤ 9), no history of psychological disorders (such as suicide, admission to psychiatry hospital, or the use of psychiatric drugs) and no cognitive disorders (such as Alzheimer’s, dementia, etc.). Exclusion criteria included readmission to the ICU, the need for mechanical ventilation after discharge, experience of severe stressful situation one month after discharge (such as death of loved ones, divorce, severe accident, etc.), GCS < 14 after discharge, unwillingness to read ICU diaries, experience of a significant stressful event after discharge, reluctance to read diaries as many times as requested by the researcher.

To determine the sample size, the mean and standard deviation was extracted from a similar study conducted by Torres et al. [14] and inserted in the formula for comparing two means. Considering the confidence interval of 95% and type II error of 95%, the sample size was estimated to be 20 patients in each group. To increase the study reliability, 30 patients were considered for each group. Finally, the data of 28 patients were analyzed (Flow diagram).

### Intervention

Patients who met the inclusion criteria were included in the study through convenience sampling method. The researcher explained them the study objectives, their full authority to discontinue the study, the information confidentiality, written consent obtained from the participants. The researcher, an ICU nurse, wrote diaries for all patients in intervention and control groups during the first 72 h of their admission to the ICU in the evening shift. It was not explained to the samples that they might be in the intervention or control group.

After the intervention, the patients were divided into intervention and control groups by using block randomization. The randomization sequence was generated using www.sealedenvelope.com. Then, ICU survivors in intervention group received the electronic version of the diaries on WhatsApp, and they were asked to read the diaries at least once, up to 3 weeks after discharge. The researcher sent a reminder text to the patients once a week up to three weeks after discharge. The researcher kept the diary related to the control group until the end of the study.

### Content of ICU diary

ICU diary includes an easy explanation of common medical terms in ICU, photos and information about the ICU environment and equipment, information about the ICU staff, information about the physical condition and course of the disease, information about diagnostic methods, the treatment and care provided for the patient, the description of the daily events and the emotional reactions of patients and their families. The researcher wrote all these information in simple words and mentioned their times and dates. In case of use of technical terms, she immediately explained them in the text. Finally, she wrote a message for the survivors wishing them to return to a healthy life with their families. Sensitive information (malignancy, HIV, sex, or substance abuse) remained confidential and was not included in the diary.

The researcher collected information from the IES-R and ICU Memory Tool one month after the patient’s discharge in order to evaluate post-traumatic stress and memories from the ICU. Data collection was done from January 2021 to August 2022.

### Blinding

**Fig. 1:**
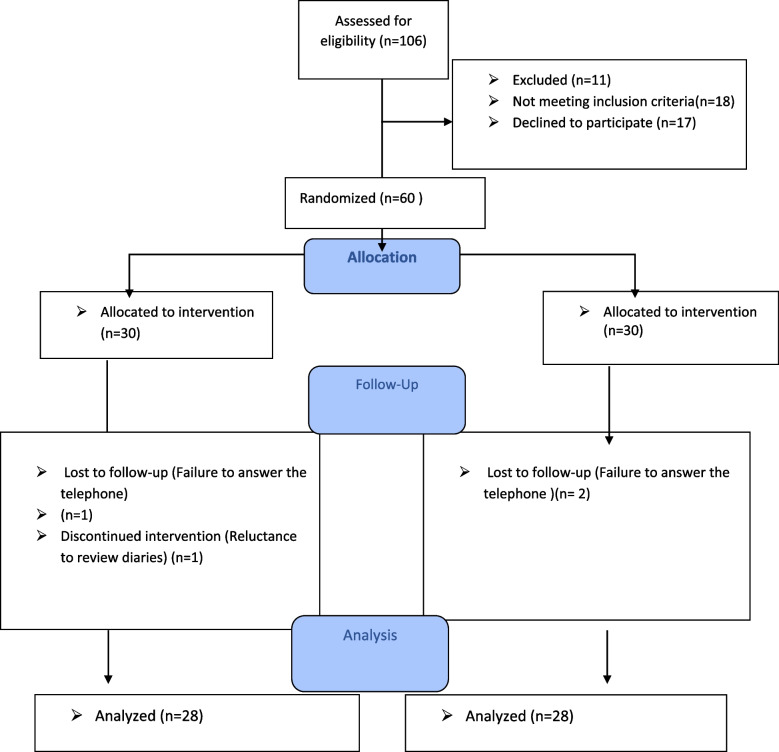
Flow diagram: the process of random allocation of the study participants

When the researcher was writing diaries, she did not assign the participants to the intervention and control groups; therefore, she was blinded to the patient group (intervention or control). The researcher who contacted them to complete the questionnaires was unaware which patients were in the intervention or control groups. The researcher who analyzed data was blinded to the groups (intervention or control) and the patients did not know their allocations into the intervention and control groups before receiving the diaries (Fig. 1).

### Instrument

Three questionnaires were used in this study: the background information, the Impact of Events Scale-Revised (IES-R), and the ICU Memories Tool (ICU-MT).

The Impact of Events Scale-Revised (IES-R) was developed by Weiss and Marmar (1997) according to the Diagnostic and Statistical Manual of Mental Disorders [15].

The IES-R is a 22-item questionnaire, of which eight items [6, 8, 9, 12, 13, 16, 17] belong to avoidance, eight items [1, 3, 4, 7, 10, 14, 18, 19] belong to intrusion, and six items [5, 11, 15, 20–22] belong to hyper arousal. Participants must complete the questionnaire according to their symptoms in the last seven days from 0 (never) to 4 (very much). Therefore, the mean scores obtained from each subscale will be between 0 and 4. The total score of the questionnaire is 0–88 [10].

Maramr and Weiss (1997) reported Cronbach’s alpha of 0.79–0.92 and test–retest correlation of 0.51-0.94. The questionnaire validity was confirmed by the factor analysis method [15].

Askari et al. (2020) translated the questionnaire into Persian, checked the reliability of the questionnaire with the internal correlation and reported Cronbach’s alpha of 82%. The content validity index (CVI) of 92% was reported in this study [10].

The ICU Memories Tool (ICU-MT) designed by Jones et al. (2000) contains eight questions. Question 4 contains 4 parts, question 6 contains 2 parts, and question 7 contains 3 parts (14 questions in total). Some questions were rated on a three-point Likert scale, including “clearly”, “vaguely” and “not at all”, some had yes and no options, and others were open-ended. One of the questions (4b) of this questionnaire divided the memories of patients from ICU into three categories:

A. Delusional memories: dreams, nightmares, delirium, the feeling that people will hurt you (4 items)
B. Factual memories: a feeding tube in the mouth and nose, family, aspiration, and medical visit (6 items)
C. Emotional memories: disappointment, fear, anxiety, depression, and pain (11 items)

The number of patients who experienced each of the memories related to question 4b was reported separately. In addition, the number of those who experienced at least one memory among the memories mentioned above was reported.

Jones et al. confirmed the construct validity of this instrument and checked its reliability using Cronbach’s alpha, which was 0.86 [18]. Askari et al. (2020) checked the validity and reliability of this questionnaire. The CVI of 95.08 and the internal consistency of 76% were calculated in this study [7].

### Statistics

SPSS25 was used to analyze the data. Descriptive statistics (absolute frequency, relative frequency, mean and standard deviation) were used to describe the demographic and background characteristics of the research units as well as post-traumatic stress and memories from the ICU in two groups. Chi-square test or Fisher’s exact test were used to check the similarity of two groups in terms of qualitative background variables. In order to examine the two groups in terms of the similarity of quantitative background variables, Mann Whitney U test was used for variables with non-normal distribution, while independent t test was used for variables with normal distribution. Mann-Whitney U test was used to compare the mean post-traumatic stress between two groups. Chi-square and Fisher’s exact tests were used to compare memories from the ICU between two groups. The distribution of information was checked by using the Kolmogorov-Smirnov test. The level of significance in this study was considered 0.05.

## Results

### Background information

As shown in Table 1, the participants in the intervention group had a mean age of 33.07 ± 11.90 years, stayed in ICU for 9.32 ± 10.58 days and were under mechanical ventilation for 6.09 ± 12.73 days, with an APACHE II score of 0.3. The participants in the control group had a mean age of 37.10 ± 14.99 years, stayed in ICU for 9.03 ± 10.47 days, and were under mechanical ventilation for 4.02 ± 7.56 days, with an APACHE II score of 6.18 ± 4.07. The results showed that the intervention and control groups were similar in background information.

**Table 1:** Comparison of background information between control and intervention groups

**Variable**		**Intervention group (*n* = 28)**	**Control group (*n* = 28)**		***p*-value**
**Age**, M (SD)		33/07 (11/90)	37/10 (14/99)	Z^b^ = -0/91	0/362
**Sex, n (%)**	Male	21	20	X^2a^ = 0/09	0/761
	Female	7	8		
**Diagnosis, n (%)**	Abdominal trauma	5	6	_	1*
	Chest trauma	9	8		
	Limb trauma	14	13		
	Spinal trauma	0	1		
**APACHEII**, M (SD)		4/64 (3/03)	6/18 (4/07)	T = -1/6	0/117
**Length of ICU stay** (days), M (SD)		9/32 (10/58)	9/03 (7/56)	Z^b^ = -0/23	0/811
**Types of mechanical ventilation, n (%)**	Invasive	14/3	32/1	X^2a^ = 2/5	0/274
	Non Invasive	21/4	14/3		
	None	64/3	53/6		
**Length of mechanical ventilation** (days), M (SD)		6/09 (12/73)	4/02 (7/56)	Z^b^ = -0/06	0/944
**Physical restraint**, n (%)	Yes	3	4	_	1*
	No	25	24		
**Benzodiazepine**, n (%)	**Yes**	4	10	X^2a^ = 42/3	0/063
	**No**	24	18		

Fishers exact test*

Chi Squared^a^

Mann-Whitney U^b^

### Posttraumatic stress disorder

As shown in Table 2, the total mean score and the mean scores of all three areas of IES-R in the intervention group were lower than that in the control group (*p* > 0.0001).

**Table 2:** Comparison of post-traumatic stress between the intervention and control groups

IES-R	Intervention group (*n* = 28)	Control group (*n* = 28)	*P*-value
Avoidance	0/29 (0/36)	0/69 (0/64)	< 0/0001
Intrusion	0/39 (0/52)	0/95 (0/67)	< 0/0001
Hyper arousal	0/36 (0/59)	0/91 (0/67)	< 0/0001
**Total score**	0/34 (0/45)	0/84 (0/62)	< 0/0001

Mann-Whitney U

### Memories

According to Table 3, the number of participants in the intervention group who clearly recalled ICU memories (82.1%) was more than that in the control group (53.6%) (*p* = 0.041). The number of participants in the intervention group (82.1%) who completely recalled their hospital stay before being admitted to the ICU was more than that in the control group (42.9%) (*P* > 0.0001). The number of patients in the intervention group who clearly recalled the entire period of ICU stay was more (92.9%) than that in the control group (48.1%) (*p* > 0.0001). The number of patients in the intervention group who felt frightened for no reason after discharge was less (17.9%) than that in the control group (46.4%) (*p* = 0.024).

**Table 3:** Comparison of recall of memories from the ICU between intervention and control groups

**Question**		**Intervention group (*n* = 28)**	**Control group (*n* = 28)**		***p*-value**
1. Do you remember being admitted to hospital?	Clearly	23	15	X^2a^ = 5/41	0/041
	Hazily	5	12		
	Not at all	0	1		
2. Can you remember the time in hospital before you were admitted to intensive care?	All of it	23	12	X^2a^ = 9/68	< 0/0001
	Some of it	5	14		
	Nothing	0	2		
3. Do you remember being in intensive care?	YES	28	27	_	1*
	NO	0	1		
4. Do you remember all of the stay clearly?	YES	26	13	X^2a^ = 13/32	< 0/0001
	NO	2	14		
5. Do you remember being transferred from intensive care to the general wards?	Clearly	28	26	_	1*
	Hazily	0	2		
	Not at all	0	0		
6. Have you had any unexplained feelings of panic or apprehension?	YES	5	13	X^2a^ = 5/2	0/024
	NO	23	15		
7. Have you had any intrusive memories from your time in hospital or of the event that led up to your admission?	YES	3	2	_	1*
	NO	25	26		
8. Have you talked about what happened to you in intensive care with:	Family	4	10	X^2a^ = 11/23	0/023
	Friend	8	8		
	Family and friend	6	9		
	Family and nurse	2	1		
	Family and friend and nurse	6	0		

Fishers exact test*

Chi Squared^a^

According to Table 4, the number of patients in the intervention group who recalled sadness (42.9%) was less than that in the control group (71.4%) (*p* = 0.03). The number of patients in the intervention group who recalled darkness (17.9%) was less than that in the control group (50%) (*p* = 0.013).

**Table 4:** Comparison of type of memories from the ICU between intervention and control groups

**Variable**			**Intervention group (*n* = 28)**	**Control group (*n* = 28)**		***p*-value**
Delusional memories	Hallucinations	Yes	2	5	_	0/429
		no	26	23		
	Nightmares	Yes	1	4	_	0/353
		no	27	24		
	Feeling that people were trying to hurt	Yes	3	5	_	0/70
		no	25	23		
Memories of feelings	Feeling down	Yes	12	20	X^2a^ = 4/6	0/034
		no	26	8		
	Feeling confused	Yes	9	6	X^2a^ = 0/82	0/365
		no	19	22		
	Pain	Yes	26	23	_	0/42
		no	2	5		
Factual memories	Family	Yes	12	18	X^2a^ = 2/58	10
		no	16	10		
	Voices	Yes	15	8	X^2a^ = 3/6	0/051
		no	13	20		
	Lights	Yes	18	19	X^2a^ = 0/08	0/779
		no	10	9		
	Breathing Tube	Yes	5	7	X^2a^ = 0/42	0/511
		no	23	21		
	Suctioning	Yes	7	2	X^2a^ = 0/76	0/384
		no	28	18		
	Darkness	Yes	5	14	X^2a^ = 6/45	0/013
		no	23	14		

Fishers exact test*

Chi Squared^a^

## Discussion

The study results suggested that the total score of post-traumatic stress in ICU survivors of the intervention group significantly reduced compared to the control group. Torres et al. (2020) and Nielsen et al. (2020) supported us and indicated that the participants of the intervention group experienced significantly less PTSD symptoms than the control group [16, 20]. These studies indicated post-traumatic stress disorder by poorly explained or disorganized memories of the traumatic situation. Therefore, filling memory gaps is one of the main reasons for the effectiveness of diaries in reducing the post-traumatic stress of these patients [21]. In a systematic review and meta-analysis, Barreto et al. (2019) indicated that the use of ICU diaries did not play a role in post-traumatic stress disorder [19]. One of the reasons for the inconsistent results of this study is the different cut points of the tools used that led to differences in the evaluation of psychological consequences. Systematic review studies used the results of qualitative studies more than the results of quantitative studies, which could affect the inconsistency of these results.

The mean scores of the avoidance, intrusion, and hyper arousal in the intervention group were significantly lower than that in the control group. This could be due to the desensitization of the processing of memories and emotions in the intervention group. According to Azizi et al. (2022), patients controlled their memories more through this intervention, and the amount of avoidance decreased in these patients [22]. Peris et al. (2011) indicated that early psychological interventions in the intensive care unit caused a significant reduction in the levels of avoidance, intrusion, and hyper arousal in the intervention group [17]. Wang et al. (2020) showed no significant difference in the IES-R score, intrusion, avoidance, number of emotional and delusional memories, and anxiety score between the groups. They pointed out that the ICU diary was not useful for the prevention of PTSD and anxiety symptoms and improvement of the quality of life of patients three months after discharge [23]. Garrouste-Orgeas et al. (2019) did not confirm the use of ICU diary for the prevention of PTSD symptoms [12]. The difference between their study and ours can be due to the difference in the method, the duration of follow-up after discharge, and the use of different tools.

We found no significant difference in recalling all kinds of memories between the intervention and control groups. Petersson et al. (2018) found that people who received diaries did not have a significant difference in recalling memories from the control group [24]. Garrouste-Orgeas et al. (2019) agreed with us and reported that this intervention did not affect the recall of memories [12]. Tripathy et al. (2022) did not support us and showed that this intervention had an effect on the memories of patients who received diaries three months after discharge from the ICU, but this intervention had no effect on patients who received the diaries one month after discharge from the ICU [25]. The difference between these findings and the previous findings may be due to the difference in the duration of the intervention and the three-month follow-up in this study. Factual memories are considered good, while delusional and emotional memories are stressful and disturbing. There were also good emotional memories, which were not evaluated in above studies, but could reduce stress and disturbing memories from the ICU [25].

Considering that the questionnaires had to be completed after the discharge, due to the dispersion of patients in the province and distance, it was not possible to access the participants in a centralized manner. In order to solve this limitation, the patient’s phone number were obtained at the time of entering the study, and the written diaries were provided to the study participants via phone or WhatsApp messenger.

## Conclusion

The results showed that the nurse-initiated diary intervention was effective in the post-traumatic stress disorder of patients admitted to the ICU. This study provided further evidence in this topic, especially in developing countries, considering the low cost of these interventions.

The results of this research can be mentioned in the form of educational workshops, in order to pay more attention to the nurse-initiated diary by nursing students and nurses and its effect on PTSD and recalling events during hospitalization of patients in ICU. We suggest medical and educational centers to use this intervention with the cooperation of clinical nurses in order to reduce the post-traumatic stress disorder in these patients. As this intervention had no significant difference in recalling various types of memories from the ICU, we recommend more studies in this field.

## Data Availability

The datasets generated and/or analysed during the current study are not publicly available, but are available from the corresponding author on reasonable request.
